# Gut microbiota–intestinal barrier crosstalk: mechanistic advances, disease relevance, and public health implications

**DOI:** 10.3389/fpubh.2026.1777910

**Published:** 2026-05-04

**Authors:** Changmei Chen, Li Zhu, Yao Huang, Yu yu Wang, Li Kong, Song Lu, Xianhui Shang

**Affiliations:** 1 Department of Physiology, Pathophysiology & Pharmacology, Medicine & Technology College of Zunyi Medical University, Zunyi, Guizhou, China; 2 Department of Pediatric Surgery, Affiliated Hospital of Zunyi Medical University, Zunyi, China

**Keywords:** chronic disease, epithelial permeability, gut microbiota, intestinal barrier, microbiota-targeted intervention, mucosal immunity, public health, short-chain fatty acids

## Abstract

**Background:**

The intestinal barrier is a critical interface between the host and the external environment, and growing evidence indicates that bidirectional crosstalk between the gut microbiota and the intestinal barrier is a key determinant of intestinal and systemic health. Disruption of this interaction has been implicated in the development of multiple chronic non-communicable diseases, including inflammatory, metabolic, neurodegenerative, and immune-mediated conditions. However, previous reviews have often examined gut microbiota or intestinal barrier dysfunction separately, with less emphasis on their bidirectional interaction as an integrated mechanistic and public health framework.

**Objective:**

This review aims to synthesize current mechanistic advances in gut microbiota–intestinal barrier crosstalk, evaluate its relevance across major disease domains, and examine its potential implications for chronic disease prevention and public health practice. In particular, this review highlights the gut microbiota–intestinal barrier axis as a unifying framework linking microbial metabolism, mucosal homeostasis, systemic inflammation, and prevention-oriented health strategies.

**Methods:**

We conducted a structured review of recent studies published between 2019 and 2025 in PubMed, Scopus, and Web of Science, with emphasis on both foundational and emerging evidence. The review focused on microbiota-derived metabolites, epithelial junction integrity, mucosal immune regulation, disease-associated barrier dysfunction, and microbiota-targeted interventions. Evidence from mechanistic, preclinical, and clinical studies was integrated to identify major advances, translational opportunities, and current limitations in the field.

**Results:**

Current evidence indicates that gut microbiota regulate intestinal barrier integrity through metabolites such as short-chain fatty acids (SCFAs), indole derivatives, and bile acids, which influence tight junction expression, mucin production, epithelial repair, and mucosal immune balance. Conversely, barrier dysfunction may promote microbial translocation, endotoxemia, and chronic low-grade inflammation, thereby contributing to diseases such as inflammatory bowel disease, type 2 diabetes, metabolic-associated fatty liver disease, and neurodegenerative or neuropsychiatric disorders. Microbiota-targeted interventions, including prebiotics, probiotics, dietary approaches, and fecal microbiota transplantation, have shown potential to restore barrier-related homeostasis. However, the current evidence remains constrained by heterogeneity in study design, incomplete causal validation, inconsistent clinical outcomes, and limited standardization of intervention strategies, all of which restrict clinical translation and large-scale public health implementation.

**Conclusion:**

The gut microbiota–intestinal barrier axis is an important determinant of health and disease and may represent a promising target for future prevention-oriented strategies. By integrating mechanistic evidence with disease relevance, translational limitations, and public health perspectives, this review provides a more coherent framework for understanding microbiota–barrier crosstalk. Future research should prioritize causal validation, standardized methodologies, and equitable implementation pathways to support the development of scalable preventive and therapeutic strategies.

## Introduction

The human gastrointestinal tract harbors a dense and diverse microbial ecosystem, collectively termed the gut microbiota, which plays a central role in maintaining host metabolic, immune, and neuroendocrine homeostasis. In recent years, growing attention has been directed toward the bidirectional crosstalk between the gut microbiota and the intestinal barrier, as this interaction is increasingly recognized as a key determinant of both intestinal and systemic health. When this dynamic equilibrium is disrupted, alterations in microbial composition, epithelial integrity, and mucosal immune regulation may interact to promote chronic inflammation and disease susceptibility (1–6). Accordingly, the gut microbiota–intestinal barrier axis has emerged as an important framework for understanding how local intestinal disturbances may contribute to broader health outcomes.

The intestinal barrier is a multilayered defense system composed of epithelial cells, mucus layers, tight junction proteins, secretory immunoglobulin A, and innate immune components. It functions not only as a physical barrier that limits microbial translocation, but also as a biologically active interface through which host tissues continuously sense and respond to microbial signals (7). Within this interface, gut microbiota and their metabolites regulate epithelial renewal, tight junction stability, mucin production, and immune homeostasis, thereby contributing to barrier resilience and host protection. Bioactive microbial metabolites, including short-chain fatty acids (SCFAs), indoles, and secondary bile acids, have been shown to influence epithelial function and mucosal signaling pathways (4, 5, 8, 9). Conversely, dysbiosis may weaken these protective mechanisms, impair barrier integrity, and facilitate the translocation of microbial products, thereby amplifying inflammatory and metabolic disturbances (10, 11). These observations indicate that the intestinal barrier should be viewed not as a passive structural boundary, but as a dynamic and responsive platform through which microbial metabolism, epithelial signaling, and host immunity are closely integrated.

The relevance of this crosstalk extends beyond gastrointestinal physiology and has important implications for chronic disease prevention and public health. Disruption of the gut microbiota–intestinal barrier axis has been associated with a wide range of chronic non-communicable diseases, including inflammatory bowel disease (IBD), type 2 diabetes, metabolic-associated fatty liver disease (MAFLD), cardiovascular disease, and neurodegenerative or neuropsychiatric disorders (1–6, 12–16). In many of these conditions, barrier dysfunction may contribute to endotoxin translocation, low-grade inflammation, immune dysregulation, and metabolic imbalance, thereby linking intestinal dysfunction to extraintestinal disease processes. From a public health perspective, these associations are particularly relevant because they connect microbiota–barrier dysfunction with highly prevalent, long-term conditions that contribute substantially to global morbidity and healthcare burden (17). This shift in understanding supports the view that the gut microbiota–intestinal barrier axis is not only a mechanistic topic in biomedical research, but also a potentially meaningful target for prevention-oriented health strategies.

Interest has therefore grown in interventions that may preserve or restore microbiota–barrier homeostasis, including prebiotics, probiotics, dietary bioactive compounds, and fecal microbiota transplantation (FMT) (18). Such approaches have been proposed as potential tools for reducing disease risk, improving host resilience, and supporting health promotion at both individual and population levels. However, despite the promise of this field, current evidence remains heterogeneous and difficult to translate into consistent clinical or public health recommendations. Differences in study design, microbial profiling methods, host background, environmental exposures, and intervention protocols continue to limit reproducibility and comparability across studies (16, 19–22). As a result, the field still lacks a sufficiently integrated framework that can connect mechanistic evidence with disease relevance and real-world preventive application.

Although previous reviews have addressed gut microbiota, intestinal permeability, or microbiome-related diseases separately, relatively few have examined gut microbiota–intestinal barrier crosstalk as a unified and bidirectional framework that links biological mechanisms with disease burden and public health relevance. Moreover, several important questions remain unresolved, including which microbial communities and metabolites are most critical for barrier regulation, how barrier dysfunction evolves during disease development, and how microbiota-targeted strategies can be translated into scalable, evidence-based, and equitable preventive interventions (23–27). In this context, the present review aims not only to summarize recent advances in gut microbiota–intestinal barrier research, but also to critically integrate current evidence by highlighting mechanistic interactions, disease relevance, translational limitations, and public health implications. By doing so, we seek to provide a more coherent and prevention-oriented overview of this rapidly evolving field and to clarify its potential significance for future chronic disease prevention and health promotion strategies.

### Mechanistic advances in gut microbiota–intestinal barrier crosstalk

**Figure 1:**
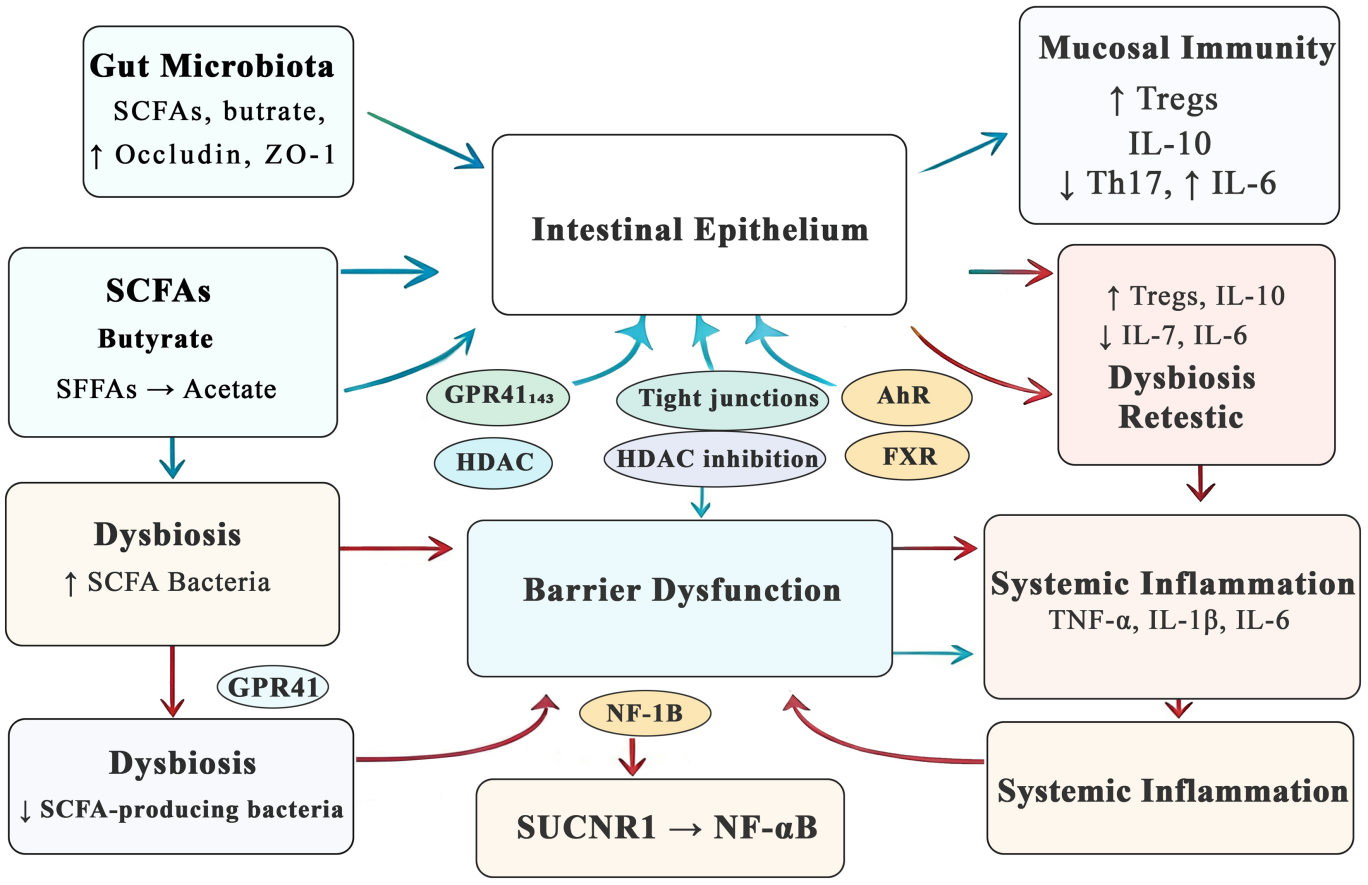
Mechanistic map of gut microbiota-intestinal barrier crosstalk. Beneficial metabolites (e.g., SCFAs) enhance tight junctions and immune tolerance. Dysbiosis and microbial toxins (e.g., LPS, succinate) impair barrier function and promote systemic inflammation.

The gut microbiota plays a critical role in maintaining both the structural and immunological integrity of the intestinal barrier. To improve conceptual clarity and avoid fragmented presentation, current mechanistic evidence may be more coherently organized into three interrelated domains: (1) beneficial microbiota-derived metabolites that preserve epithelial and immune homeostasis, (2) dysbiosis-associated signals that impair barrier function and promote inflammation, and (3) emerging microbiota-targeted interventions together with their translational limitations. This integrated framework is consistent with the broader view that gut microbiota–intestinal barrier crosstalk is a dynamic and bidirectional process with both mechanistic and public health relevance (Figure 1).

Among the protective mechanisms identified to date, microbiota-derived metabolites remain the best-characterized mediators of barrier maintenance. Accumulating evidence from mechanistic and multi-omics studies has demonstrated that short-chain fatty acids (SCFAs), particularly butyrate, propionate, and acetate, serve as key regulators of epithelial homeostasis. These metabolites enhance the expression of tight junction proteins, including claudin-1, occludin, and ZO-1, thereby strengthening epithelial integrity (4, 5, 28). Beyond their effects on junctional architecture, SCFAs also support epithelial energy metabolism, promote mucus layer maintenance, and contribute to immune tolerance within the intestinal microenvironment, collectively improving barrier resilience (29, 30). Mechanistically, SCFAs exert their protective actions through activation of G-protein–coupled receptors, especially GPR41 and GPR43, and through inhibition of histone deacetylases (HDACs), thereby regulating transcriptional programs involved in epithelial repair and anti-inflammatory signaling (31, 32). They also promote regulatory T cell (Treg) differentiation and suppress Th17-associated inflammatory responses, further supporting mucosal immune homeostasis (7). Taken together, these findings indicate that SCFAs should not be regarded merely as microbial metabolic by-products, but as active signaling mediators linking microbial activity to epithelial stability and immune regulation.

Additional microbiota-derived metabolites further extend this protective network. Indole derivatives and secondary bile acids have been shown to contribute to epithelial renewal, mucin production, and immune regulation through pathways involving the aryl hydrocarbon receptor (AhR), farnesoid X receptor (FXR), and related signaling mechanisms (33, 34). These observations suggest that barrier preservation depends on a coordinated network of metabolite-mediated signals rather than on any single molecular pathway. This broader perspective is important because it highlights the functional redundancy and complexity of microbiota–barrier regulation, while also helping to explain why disruption of microbial ecology may have widespread downstream consequences.

In contrast, dysbiosis-associated alterations in microbial composition and metabolite profiles may actively weaken these protective mechanisms and drive barrier dysfunction. Emerging evidence suggests that impaired microbiota–barrier communication can increase intestinal permeability, facilitate endotoxin translocation, amplify low-grade inflammation, and disturb immune–metabolic homeostasis, thereby extending intestinal dysfunction to extraintestinal disease processes (35–34). For example, the accumulation of microbial metabolites such as succinate has been shown to enhance colonic inflammation through activation of the SUCNR1/NF-κB signaling pathway, thereby exacerbating epithelial barrier dysfunction and promoting systemic inflammatory responses (11). At the same time, dysbiosis may reduce the production of beneficial metabolites such as SCFAs and indole derivatives, thereby weakening epithelial repair capacity, impairing tight junction stability, and amplifying mucosal immune dysregulation (32, 33). Accordingly, altered metabolite profiles should be interpreted not simply as downstream indicators of microbial imbalance, but as functional drivers of barrier deterioration and chronic inflammatory activation (34).

Barrier homeostasis is also shaped by specialized epithelial and immune cell populations, further underscoring the context-dependent nature of microbiota–barrier interactions. GP2-expressing microfold (M) cells have emerged as key mediators of mucosal immune surveillance. Notably, pasteurized *Akkermansia muciniphila* has been reported to upregulate GP2 expression, thereby enhancing antigen sampling while potentially increasing susceptibility to enteric pathogens such as *Salmonella* (30). This example illustrates that microbiota-mediated barrier regulation cannot be categorized as uniformly beneficial or uniformly harmful. Rather, its biological consequences depend on host immune status, microbial context, and the balance between immune surveillance and epithelial vulnerability (35). Such context dependence should be carefully considered when interpreting mechanistic findings and when evaluating the translational potential of microbiota-directed therapies.

In parallel with these mechanistic observations, early intervention studies suggest that microbiota–barrier interactions may be therapeutically modifiable. Herbal formulations and nutraceutical interventions have shown potential in this regard. For example, Pingwei Powder has been reported to alleviate high-fat diet–induced inflammation by restoring SCFA levels and reinforcing epithelial junction integrity, while Sishen Pill combined with sodium propionate appears to enhance mucosal healing and tight junction protein expression (29, 31). These findings support the possibility that barrier restoration may be achieved through coordinated metabolic and immunological modulation. However, the available evidence remains largely preclinical, and the mechanistic specificity, reproducibility, and population-level generalizability of these approaches remain insufficiently defined (33, 34). Their clinical and public health relevance should therefore be interpreted cautiously until supported by larger, better-controlled, and more reproducible studies.

Despite substantial progress, the current mechanistic literature remains constrained by important methodological and translational limitations. Most available evidence is still derived from experimental systems, whereas direct causal validation in human populations remains limited. In addition, differences in microbial composition, host background, dietary exposure, disease context, intervention protocol, and analytical platform continue to hinder cross-study comparison and reduce translational applicability (38, 39). This uneven evidence base makes it difficult to determine which pathways are consistently reproducible, clinically meaningful, and suitable for prevention-oriented application. For this reason, future studies should move beyond descriptive association and prioritize evidence hierarchy, including reproducible mechanistic validation, well-designed human studies, cross-cohort comparison, and standardized multi-omics frameworks for mechanistic and translational investigation (35).

To further synthesize the current evidence, Table 1 summarizes representative microbial metabolites, their microbial origins, major signaling pathways, and their reported effects on intestinal barrier structure and function.

**Table 1:** Representative microbiota-derived metabolites, signaling pathways, and their roles in intestinal barrier regulation.

Metabolite/microbial factor	Representative microbial source	Major signaling pathway	Principal effect on barrier function	Ref.
Butyrate	*Faecalibacterium prausnitzii*	GPR43, HDAC inhibition	↑ Tight junction proteins (ZO-1, claudin-1), anti-inflammation	(4, 5, 28, 29)
Succinate	*Bacteroides* spp.	SUCNR1	Activates NF-κB, ↑ inflammation, ↓ epithelial integrity	(4, 11)
Indole derivatives	*Lactobacillus*, *Clostridium* spp.	AhR	↑ Mucin secretion, ↑ Treg/Th17 balance	(32, 41)
Secondary bile acids	Firmicutes, Clostridia	FXR, TGR5	Modulate TLRs, mucosal immunity	(5, 33)
Lipopolysaccharide	Gram-negative bacteria	TLR4	↑ Systemic inflammation, barrier disruption	(20, 34)
Polyamines	*E. coli*, *Bifidobacteria*	—	Promote epithelial proliferation and mucus layer	(5, 35)

Overall, the available evidence supports a dynamic, bidirectional, and context-dependent model in which commensal bacteria and their metabolites reinforce epithelial integrity and mucosal immune balance, whereas dysbiosis and microbiota-derived harmful signals, such as lipopolysaccharide (LPS) and succinate, disrupt barrier function and promote permeability, immune dysregulation, and systemic inflammation. At the same time, substantial inter-individual variability, environmental exposures, and methodological heterogeneity continue to shape these mechanisms and may account for inconsistency across studies. Consequently, further longitudinal, multi-omics–integrated, and clinically oriented investigations are required to strengthen causal inference, improve reproducibility, and enhance the translational and public health relevance of microbiota–barrier research.

### Disease relevance of gut barrier disruption

Gut barrier dysfunction is increasingly recognized as a shared and integrative mechanism underlying a broad range of chronic diseases, extending beyond localized intestinal pathology to systemic disorders with major clinical and public health relevance (4–6, 33). Rather than representing an isolated gastrointestinal abnormality, impaired barrier integrity should be understood as a cross-disease pathophysiological interface linking microbial imbalance, immune dysregulation, metabolic disturbance, and inter-organ communication. To improve conceptual clarity, the current evidence can be grouped into three major domains: intestinal inflammatory diseases, metabolic and cardiometabolic disorders, and neuropsychiatric or neurodegenerative conditions. This organization better reflects the common downstream consequences of barrier disruption while also highlighting differences in the strength and maturity of the available evidence.

The strongest mechanistic and translational evidence currently comes from intestinal inflammatory disorders, particularly inflammatory bowel disease (IBD). In IBD, dysbiosis and the depletion of short-chain fatty acid (SCFA)-producing taxa impair epithelial integrity, promote microbial translocation, and trigger aberrant immune activation (4, 5, 33, 34). These processes are further reinforced by metabolite-driven inflammatory signaling and altered host–microbiome interactions, as supported by recent multi-omics investigations (23–25). Compared with other disease categories, the IBD literature provides relatively consistent support for a pathogenic link between barrier dysfunction and disease activity, although the directionality and temporal sequence of these events may still vary across patient subgroups and disease stages.

A second major group includes metabolic and cardiometabolic diseases, in which gut barrier disruption appears to contribute to systemic endotoxemia, chronic low-grade inflammation, and metabolic dysregulation. Increased gut permeability has been observed in patients with type 2 diabetes, where translocation of lipopolysaccharide (LPS) and other microbial products may contribute to insulin resistance and hepatic steatosis through the gut–liver axis (2, 13). Similarly, disruption of barrier integrity has been implicated in metabolic-associated fatty liver disease (MAFLD) and related conditions, where bacterial endotoxins and altered microbial signaling may promote hepatic inflammation, lipid accumulation, and fibrotic progression (4, 5, 13, 35). In cardiovascular disease, gut-derived inflammatory signals, particularly LPS-mediated vascular activation, have also been linked to endothelial dysfunction and atherosclerotic progression (20, 34). Collectively, these findings support a broader model in which barrier disruption contributes to metabolic and vascular disease through interconnected inflammatory and immune–metabolic pathways.

However, the clinical interpretation of these associations remains more complex than in classical intestinal disorders. Inter-individual variability in microbiome composition, host susceptibility, dietary exposure, and environmental context may partly explain the inconsistency observed across studies (21, 22). Accordingly, the disease relevance of gut barrier dysfunction in metabolic disorders should not be inferred solely from the presence of dysbiosis or increased permeability, but should instead be interpreted within a broader framework that includes host background, metabolic state, and exposure-related modifiers. This distinction is particularly important for prevention-oriented research, where mechanistic plausibility alone is insufficient to justify population-level application.

A third and increasingly studied domain involves neuropsychiatric and neurodegenerative disorders, including Alzheimer’s disease, Parkinson’s disease, and psychosis-related conditions. Emerging evidence suggests that gut barrier dysfunction may facilitate the translocation of microbial metabolites, endotoxins, and pro-inflammatory cytokines, thereby contributing to neuroinflammation, neurotransmitter dysregulation, and pathological protein aggregation (15, 16, 36–40). Within this framework, the gut–brain axis represents a bidirectional communication network involving neural, endocrine, and immune pathways. Experimental studies further indicate that microbiota modulation, including fecal microbiota transplantation and probiotic interventions, can influence behavior, cognitive outcomes, and blood–brain barrier integrity (15, 37). Nevertheless, compared with IBD and some metabolic disorders, the clinical evidence in this area remains more heterogeneous and often less causally resolved.

This heterogeneity has important implications for interpretation. Although current findings support the possibility that gut barrier dysfunction contributes to brain disorders through inflammatory spillover and broader disturbances in neuroimmune and neuroendocrine signaling, the relative contribution of barrier dysfunction compared with other disease drivers remains uncertain. Therefore, this field should be viewed as promising but still evolving, with a need for greater caution in translating preclinical observations into clinical or public health claims.

Taken together, the available evidence supports a systems-level view in which intestinal barrier integrity influences disease processes across multiple organ systems, including the intestine, liver, vasculature, and brain. From a public health perspective, this pattern is particularly important because it suggests that gut barrier dysfunction may represent a shared upstream pathway contributing to several highly prevalent non-communicable diseases. At the same time, the evidence is not equally robust across all disease domains. The strongest support currently comes from inflammatory bowel disorders and selected metabolic conditions, whereas the evidence for neuropsychiatric and some extraintestinal outcomes remains more variable in quality and consistency.

Despite substantial progress, several limitations continue to constrain interpretation and translation. Current evidence remains affected by heterogeneity in study design, population-specific differences, diagnostic criteria, microbiome profiling platforms, and outcome measures. These factors hinder cross-study comparison and may partly account for conflicting conclusions in the literature. In addition, causal relationships remain difficult to establish, particularly in human studies where barrier dysfunction may act as both a contributor to and a consequence of disease progression. For this reason, future research should prioritize longitudinal, multi-omics–integrated, and clinically stratified investigations to clarify temporal relationships, improve reproducibility, and strengthen the evidence base for precision prevention and intervention strategies.

**Figure 2:**
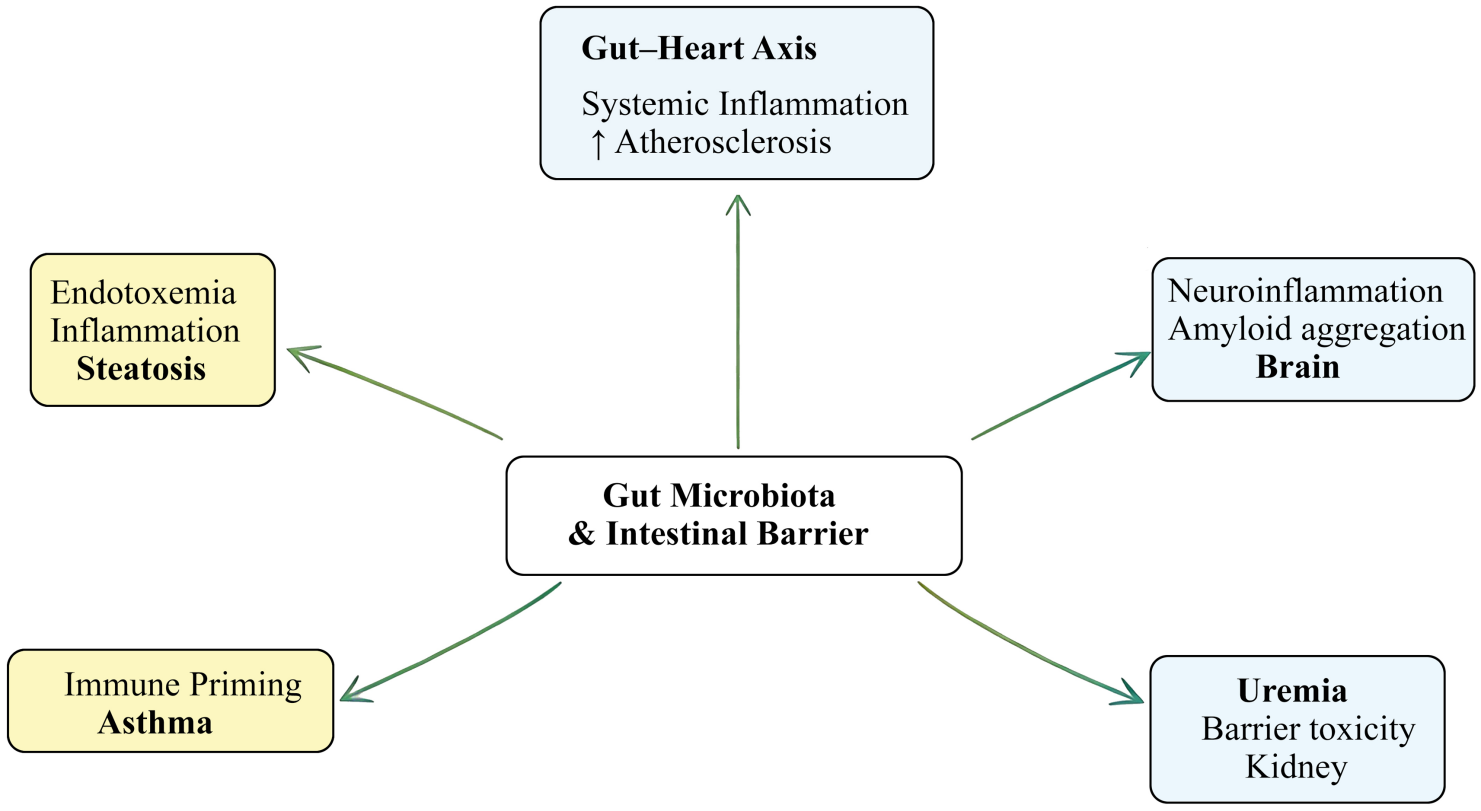
Gut-organ axis model illustrating how intestinal barrier dysfunction contributes to systemic chronic diseases via microbial and immune signaling.

As illustrated in Figure 2, multiple organ systems—including the brain, liver, lung, and vasculature—are influenced by gut-derived signals, forming interconnected axes through which intestinal barrier integrity may shape systemic disease outcomes. To further synthesize the current evidence, Table 2 summarizes representative disease associations, major mechanisms of barrier disruption, principal consequences, and the general level of evidence supporting each association.

**Table 2:** Representative disease associations of gut barrier dysfunction, underlying mechanisms, and general level of evidence.

Disease	Representative mechanism of barrier disruption	Principal consequence	General level of evidence	Ref.
Inflammatory bowel disease (IBD)	Dysbiosis, reduced SCFA production, and impaired tight junction integrity	Mucosal inflammation and disease exacerbation	Relatively strong mechanistic and clinical support	(4, 33–38)
Type 2 diabetes (T2D)	Increased LPS translocation and metabolic endotoxemia	Insulin resistance and hepatic steatosis	Moderate clinical and mechanistic support	(2, 5, 13, 22)
Alzheimer’s disease	Leaky gut, microbial metabolite translocation, and neuroinflammatory signaling	Neuroinflammation and pathological protein aggregation	Emerging but heterogeneous evidence	(15, 24, 32, 39, 36, 37, 39, 41)
NAFLD/MAFLD	Gut-liver axis disruption and increased bacterial endotoxin exposure	Hepatic inflammation, steatosis, and fibrosis	Moderate mechanistic and translational support	(4, 5, 13, 35)
Atherosclerosis	Gut-derived LPS and vascular endothelial activation	Plaque progression and systemic inflammation	Emerging to moderate evidence	(20, 34)
Chronic kidney disease (CKD)	Uremic toxin accumulation, dysbiosis, and epithelial atrophy	Systemic toxin burden and immune dysfunction	Limited but suggestive evidence	(21, 35)

### Public health implications and preventive opportunities

The public health relevance of gut microbiota–intestinal barrier dysfunction lies in its potential contribution to a wide range of chronic non-communicable diseases that account for substantial global morbidity, long-term healthcare burden, and growing preventive challenges, particularly in aging and urbanized populations (4–6, 17, 18). As evidence accumulates linking microbiota–barrier disruption to inflammatory, metabolic, neurocognitive, and immune-mediated conditions, the field is increasingly moving beyond a purely mechanistic focus toward a prevention-oriented framework. Within this framework, preservation of barrier integrity is viewed not only as a therapeutic target, but also as a potential upstream strategy for reducing disease risk and improving population health. This shift is further supported by large-scale population studies and multi-omics investigations highlighting the central role of host–microbiome interactions in disease susceptibility and progression (23–25).

At the intervention level, nutritional and microbiota-targeted strategies have received growing attention because they may offer relatively scalable approaches to barrier preservation and risk reduction. Prebiotic fibers, dietary polyphenols, fermented foods, and related dietary approaches have shown potential to modulate microbial composition and enhance barrier function (5, 22, 35). In parallel, more intensive microbiota-directed interventions, including fecal microbiota transplantation (FMT) and next-generation probiotics, are being explored in randomized or translational studies involving ulcerative colitis, type 1 diabetes, and autism spectrum disorders (37). However, despite this growing interest, current outcomes remain heterogeneous and often difficult to generalize. Variability in baseline microbiota composition, host genetics, environmental exposure, intervention protocol, and study design continues to limit reproducibility and cross-study comparability (21, 22). These limitations suggest that microbiome-informed interventions, although promising, cannot yet be translated directly into broad public health recommendations without stronger causal evidence, more consistent efficacy, and better-defined implementation contexts.

From a public health policy perspective, translating microbiota–barrier science into practice requires a systems-based rather than disease-specific approach. Potential strategies include incorporating gut health concepts into evidence-based dietary guidance, strengthening public education on microbiota-supportive lifestyles, improving food labeling practices where scientifically justified, and supporting surveillance or research infrastructures relevant to microbial risk markers. At the same time, caution is necessary to avoid premature overgeneralization, because the current evidence base remains uneven across interventions, populations, and disease settings. Accordingly, the public health value of this field lies not simply in proposing new microbiota-targeted products or treatments, but in identifying which aspects of microbiota–barrier knowledge can be translated into realistic, equitable, and evidence-based prevention strategies.

**Figure 3:**
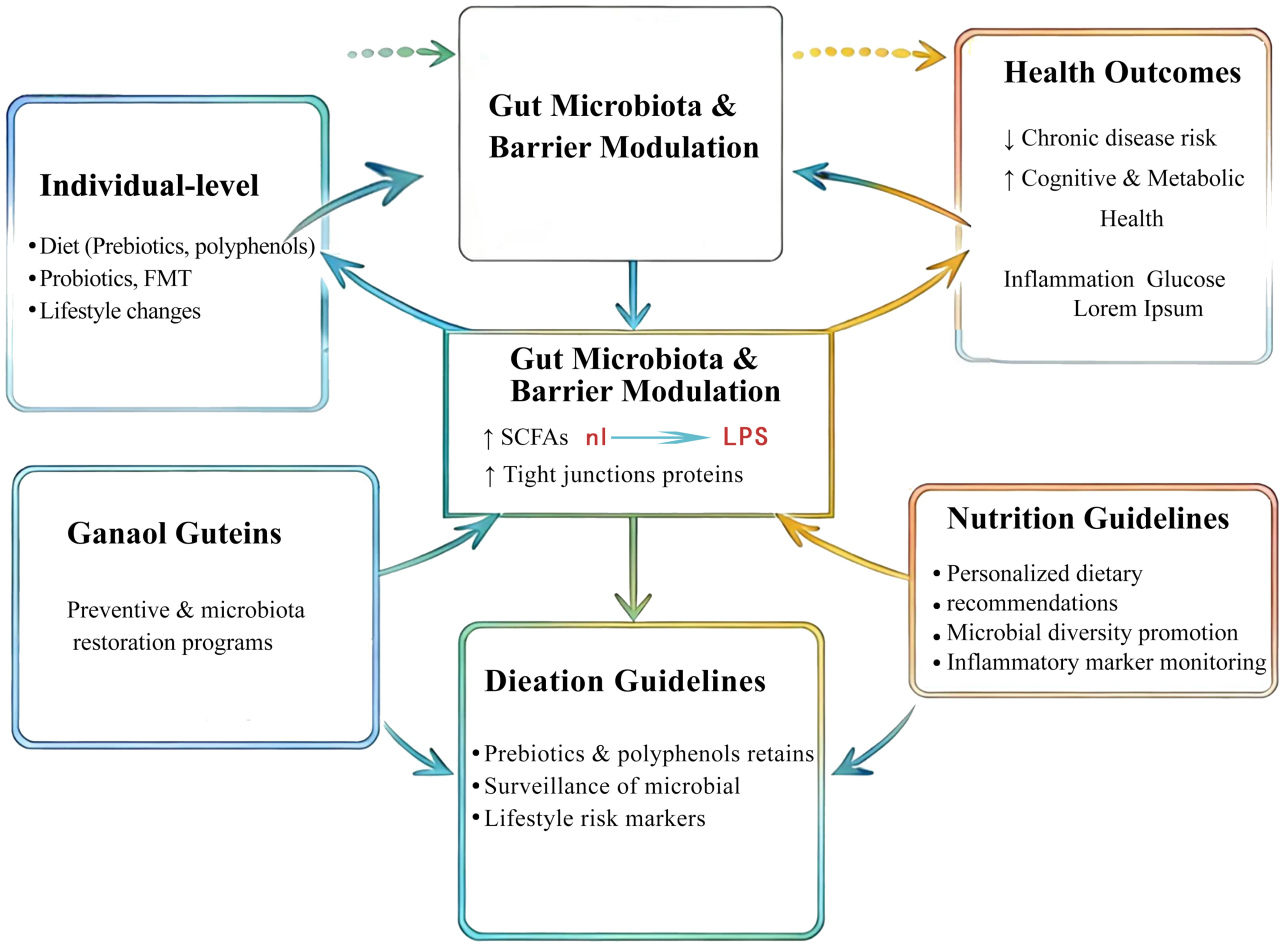
Translational framework from individual microbiota-barrier interventions to population-level public health strategies targeting chronic disease prevention.

As illustrated in Figure 3, microbiota–intestinal barrier science may be conceptualized within a multilevel public health framework that spans individual, community, and policy domains. At the individual level, relevant strategies include microbiome-supportive diets, lifestyle modification, and selected over-the-counter synbiotic approaches. At the community level, health education campaigns, workplace wellness initiatives, and front-of-pack gut health communication may improve awareness and behavior where evidence supports such measures. At the policy or system level, broader actions may include the integration of microbiota-related considerations into national dietary frameworks, investment in microbiome research, and the development of surveillance approaches for microbial and metabolic risk patterns. Rather than focusing solely on downstream treatment, this framework emphasizes upstream prevention, risk reduction, and cross-sector coordination.

Nevertheless, substantial barriers remain before microbiome-informed prevention can be implemented at scale. Future efforts should prioritize causal inference, longitudinal cohort validation, regulatory standardization, and equitable implementation across diverse populations and socioeconomic settings. Greater attention should also be given to accessibility, population heterogeneity, and the risk of widening health disparities if microbiota-based strategies are developed without adequate public health oversight. These considerations are essential if microbiome science is to move from promising concept to sustainable and socially relevant public health practice.

## Conclusion

The gut microbiota–intestinal barrier axis has emerged as an important determinant of systemic health and disease vulnerability, with implications extending from mechanistic biology to chronic disease prevention and public health strategy. Mechanistic insights into microbial metabolites, immune signaling, and epithelial structure have revealed potential therapeutic and preventive targets across a range of chronic conditions. Importantly, this review emphasizes that microbiota–barrier crosstalk should not be viewed merely as a localized gastrointestinal phenomenon, but as a broader integrative framework linking microbial homeostasis, immune regulation, metabolic disturbance, and multi-organ disease processes.

At the same time, the current evidence base remains constrained by substantial heterogeneity in study design, population characteristics, microbiome profiling methodology, and intervention protocols. These limitations continue to restrict causal inference, reproducibility, and the comparability of findings across studies. In particular, inconsistent results from microbiota modulation strategies, including probiotics and fecal microbiota transplantation, indicate that translation into routine clinical and public health practice is still at an early stage. Accordingly, enthusiasm for this field should be balanced by careful attention to evidence quality, context specificity, and implementation feasibility.

Future research should prioritize multi-omics integration, longitudinal cohort validation, and mechanistic stratification of relevant population subgroups in order to strengthen translational precision. Equally important, bridging experimental evidence with real-world prevention requires scalable, evidence-based, and equitable public health frameworks, as summarized in Table 3. Greater emphasis should also be placed on cross-cohort reproducibility, standardized analytical and reporting frameworks, and clinically or population-relevant validation.

**Table 3:** Representative public health strategies targeting the gut microbiota–barrier axis.

Intervention level	Representative strategies	Primary mechanistic target	Intended public health outcome	Evidence reference
Individual level	Prebiotics, fermented foods, SCFA-supportive dietary strategies	Improved SCFA production and maintenance of tight junction integrity	Improved microbiota diversity and reduced gut permeability	(5, 22, 35)
Community level	Gut health education, front-of-pack communication, workplace wellness initiatives	Diet–microbiota modulation and health behavior change	Improved awareness and healthier behavior patterns	(21, 42)
Policy/system level	Dietary guidance, microbiome research funding, surveillance and prevention frameworks	Population-level microbiota optimization and early risk monitoring	Reduced NCD burden through upstream prevention	(17, 23–25, 40)

Overall, the preservation of gut barrier integrity may become an important component of future preventive medicine. By integrating mechanistic evidence, disease relevance, translational limitations, and public health priorities, this field provides a promising—although still evolving—foundation for more precise, preventive, and system-oriented approaches to chronic disease control.
